# Attenuation of Ascites Following Surgical Ligation of a Congenital Caudal Mesenteric Arteriovenous Fistula in a Dog

**DOI:** 10.1155/crve/8275438

**Published:** 2026-04-16

**Authors:** Shuji Suzuki, Sachiyo Tanaka, Nobuo Kanno, Takuya Yogo, Yasuji Harada, Masaki Michishita, Yasushi Hara

**Affiliations:** ^1^ Laboratory of Veterinary Surgery, Faculty of Veterinary Science, School of Veterinary Medicine, Nippon Veterinary and Life Science University, Musashino, Japan, nvlu.ac.jp; ^2^ Laboratory of Veterinary Pathology, Faculty of Veterinary Science, School of Veterinary Medicine, Nippon Veterinary and Life Science University, Musashino, Japan, nvlu.ac.jp

## Abstract

A 1‐year‐and‐9‐month‐old intact female Pembroke Welsh Corgi (10.5 kg) was referred for abdominal distension, intermittent diarrhea, and ascites. Abdominocentesis yielded 1200 mL of transudate (specific gravity, 1.008). Serum biochemistry showed hyperammonemia (NH_3_, 163 *μ*g/dL), increased bile acids, and mild hypoalbuminemia. Abdominal ultrasonography suggested a vascular anomaly, and contrast‐enhanced computed tomographic angiography confirmed an arteriovenous communication between the caudal mesenteric artery and vein with marked venous dilation and multiple acquired portosystemic shunts, consistent with portal hypertension secondary to a congenital caudal mesenteric arteriovenous fistula. Surgical ligation was performed via ventral midline celiotomy. Portal venous pressure measured via a mesenteric vein decreased from 23/20 (21) mmHg to 19/16 (17) mmHg after occlusion; a 5‐min test occlusion caused no gross intestinal congestion. Liver biopsy revealed portal vein hypoplasia with reduced portal triads. Ascites resolved by Postoperative Day 3, and the dog was discharged on Postoperative Day 6 (Day 22). Ascites recurred on Day 868, but follow‐up computed tomography on Day 1191 confirmed persistent occlusion of the fistula. Under adjunct medical management, the dog remained clinically stable with only mild hyperammonemia (NH_3_, 132 *μ*g/dL). This case suggests that surgical ligation can reduce portal hypertension and resolve ascites in dogs with caudal mesenteric arteriovenous fistulas, although long‐term monitoring may be required.

## 1. Introduction

Arteriovenous fistulas involving the mesenteric vasculature are extremely rare in dogs [1, 2]. These arterioportal communications shunt arterial blood directly into the portal venous system, increasing portal venous inflow and resulting in portal hypertension [3, 4]. Consequently, affected dogs may develop secondary acquired portosystemic shunts, gastrointestinal signs, hyperammonemia, and, in severe cases, ascites [4–6]. Computed tomographic (CT) angiography can be useful for characterizing the vascular anatomy and classifying abdominal arterioportal communications in dogs and cats [7]. In previously reported canine cases, mesenteric arteriovenous/arterioportal fistulas have been described predominantly in association with the cranial mesenteric vasculature [1, 2]. To the authors’ knowledge, involvement of the caudal mesenteric vasculature has not been previously reported in dogs. Because this condition is exceptionally uncommon, information regarding optimal diagnostic approaches, therapeutic strategies, and long‐term outcomes is limited.

Surgical interruption of the abnormal arteriovenous communication has been proposed as a rational treatment option because it directly reduces excessive arterial inflow into the portal venous system [4]. However, published clinical data supporting surgical ligation for congenital mesenteric arteriovenous fistulas in dogs remain limited, particularly with respect to objective assessment of portal venous pressure and long‐term outcome [1, 2]. The purpose of this report is to describe the clinical presentation, diagnostic imaging findings, surgical management, and long‐term outcome of a dog with a congenital caudal mesenteric arteriovenous fistula. This case highlights the potential effectiveness of surgical ligation in reducing portal hypertension and resolving ascites in dogs.

## 2. Case Presentation

A 1‐year‐and‐9‐month‐old intact female Pembroke Welsh Corgi weighing 10.5 kg was referred to the Animal Medical Center of Nippon Veterinary and Life Science University for further evaluation and treatment of ascites. Approximately 2 months prior to presentation, progressive abdominal distension and intermittent diarrhea had been noted by the referring veterinarian. Protein‐losing enteropathy was suspected, and medical management was initiated; however, the clinical signs did not improve. The dog was therefore referred for further investigation and management of persistent ascites.

On physical examination at the initial visit (Day 1), marked abdominal distension was observed. Abdominal ultrasonography revealed a large volume of free abdominal fluid. To alleviate abdominal tension, abdominocentesis was performed, and approximately 1200 mL of clear, low‐specific gravity fluid was removed. The ascitic fluid had a specific gravity of 1.008, total protein of 2.8 g/dL, and a total nucleated cell count of 500/*μ*L; cytology revealed no clinically significant abnormalities and was consistent with a transudate.

Hematologic and coagulation testing performed at the initial presentation (Day 1) revealed no clinically relevant abnormalities (Table 1). Serum biochemical analysis showed hyperammonemia (NH_3_, 163 *μ*g/dL) and increased total bile acids (22.2 *μ*mol/L), accompanied by mild hypoalbuminemia (2.2 g/dL). Mild decreases in serum sodium (128 mmol/L) and chloride (97 mmol/L) concentrations were also noted (Table 2).

**Table 1:** Hematologic and coagulation findings at initial presentation (Day 1).

Parameter	Result	Reference interval	Unit
RBC	6.29	5.50–8.50	×10^6^/*μ*L
Hb	10.3	12.0–18.0	g/dL
PCV	37.8	37.0–55.0	%
MCV	60.1	60.0–77.0	fL
MCHC	34.7	32.0–36.0	g/dL
Platelets	196	200–500	×10^3^/*μ*L
WBC	7900	6000–17,000	/*μ*L
PT	6.9	6.0–9.5	s
APTT	15	9.0–16.5	s
Fibrinogen (FBG)	254	150–350	mg/dL
Antithrombin III (ATIII)	139.6	70–160	%
FDP	2.9	0.0–4.0	*μ*g/mL
D‐dimer	1.4	0.0–2.0	*μ*g/mL

**Table 2:** Serum biochemical and electrolyte findings at initial presentation (Day 1).

Parameter	Result	Reference interval	Unit
Ammonia (NH₃)	163	< 75	*μ*g/dL
Total bile acids (TBAs)	22.2	0.3–20	*μ*mol/L
Glucose	88	75–128	mg/dL
Total protein	5	4.9–7.2	g/dL
Albumin	2.2	2.0–3.2	g/dL
ALT	50	14–68	U/L
AST	34	14–44	U/L
ALP	157	47–254	U/L
BUN	6.7	9.2–29.2	mg/dL
Creatinine	0.4	0.40–1.45	mg/dL
CK	94	47–168	U/L
Phosphorus (P)	4.3	1.9–5.0	mg/dL
Calcium (Ca)	9.2	9.1–12.3	mg/dL
Sodium (Na)	128	141–152	mmol/L
Potassium (K)	4.8	3.8–5.1	mmol/L
Chloride (Cl)	97	102–117	mmol/L
CRP	0.7	0.0–1.0	mg/dL
Total cholesterol (T‐Cho)	137	105–322	mg/dL
Triglycerides (TG)	19	17–113	mg/dL
BCAA	194	325–825	*μ*mol/L
Tyrosine (TYR)	142	25.5–83.5	*μ*mol/L
BTR	1.4	4.0–25.0	—

Abbreviations: APTT, activated partial thromboplastin time; ATIII, antithrombin III; BCAA, branched‐chain amino acids; BTR, branched‐chain amino acid‐to‐tyrosine ratio; FDP, fibrin/fibrinogen degradation products; Hb, hemoglobin; ICT, icterus index; MCHC, mean corpuscular hemoglobin concentration; MCV, mean corpuscular volume; PCV, packed cell volume; PT, prothrombin time; RBC, red blood cell count; TBA, total bile acids; TP, total protein; WBC, white blood cell count.

Abdominal ultrasonography performed at the initial presentation revealed a large volume of ascites and microhepatia (Figure 1a). In addition, an abnormally dilated vessel was identified in the region adjacent to the urinary bladder, with apparent arterial inflow and turbulent flow on color Doppler imaging, raising suspicion of a congenital vascular anomaly (Figure 1b). A urine protein‐to‐creatinine ratio was not obtained; however, abdominal ultrasonography showed no renal or gastrointestinal abnormalities suggestive of protein‐losing nephropathy or protein‐losing enteropathy. Thoracic radiography and echocardiography were performed and revealed no abnormalities consistent with right‐sided congestive heart failure.

Figure 1Abdominal ultrasonographic findings at initial presentation. (a) B‐mode image showing a large volume of anechoic peritoneal effusion surrounding the spleen (ascites). (b) Color Doppler image obtained in the region adjacent to the urinary bladder demonstrating an abnormally dilated vessel with turbulent, mosaic flow consistent with arterialized inflow, suggesting a congenital vascular anomaly (arrow).

**Figure:**
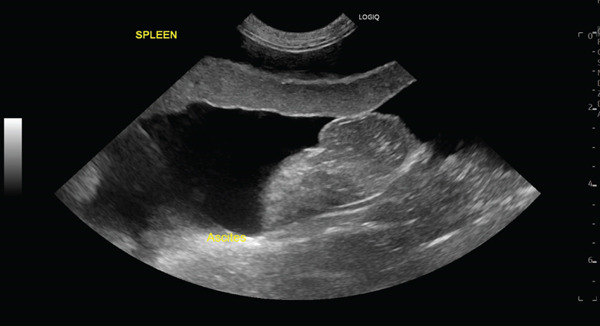
(a)

**Figure:**
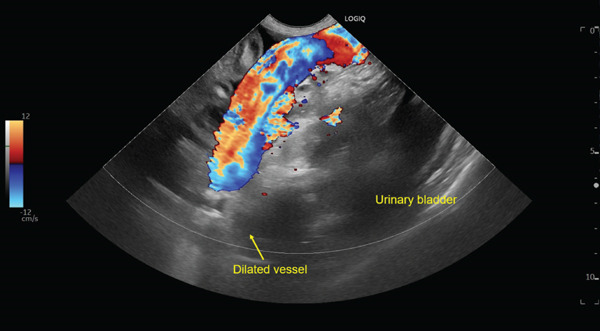
(b)

Contrast‐enhanced CT angiography was performed on Day 9. CT angiography demonstrated an arteriovenous communication between the caudal mesenteric artery and vein, marked dilation of the draining vein, and multiple acquired portosystemic shunts (Figure 2a,b and Video S1).

Figure 2Three‐dimensional volume‐rendered CT angiographic images of the abdominal viscera and associated vasculature. (a) Coronal CT angiographic image demonstrating marked dilation of the caudal mesenteric vein. (b) Sagittal CT angiographic image demonstrating direct communication between the caudal mesenteric artery and caudal mesenteric vein, confirming a caudal mesenteric arteriovenous fistula. The asterisk indicates the site of the arteriovenous fistula. Abbreviations: CdMA = caudal mesenteric artery, CdMV = caudal mesenteric vein, RK = right kidney, LK = left kidney.

**Figure:**
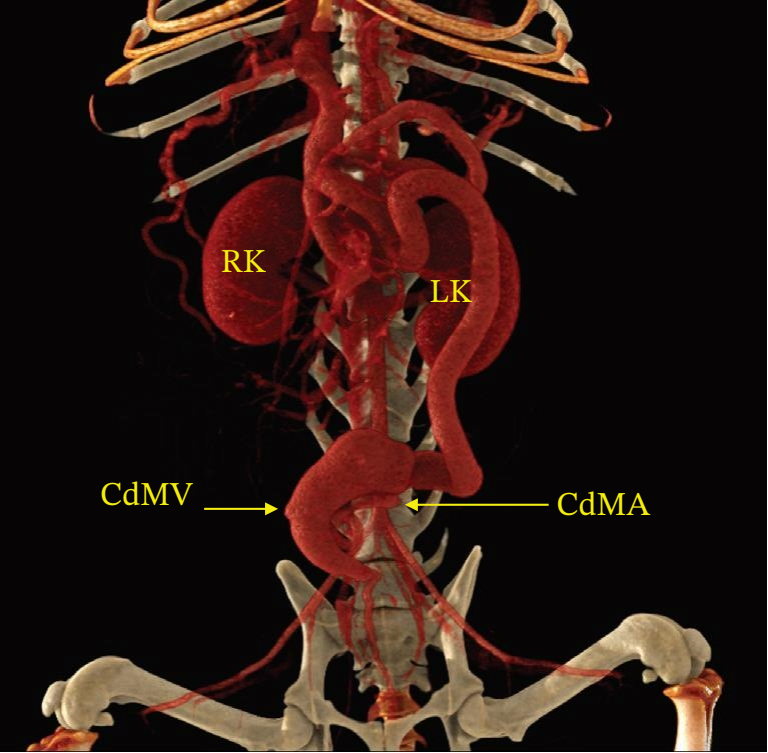
(a)

**Figure:**
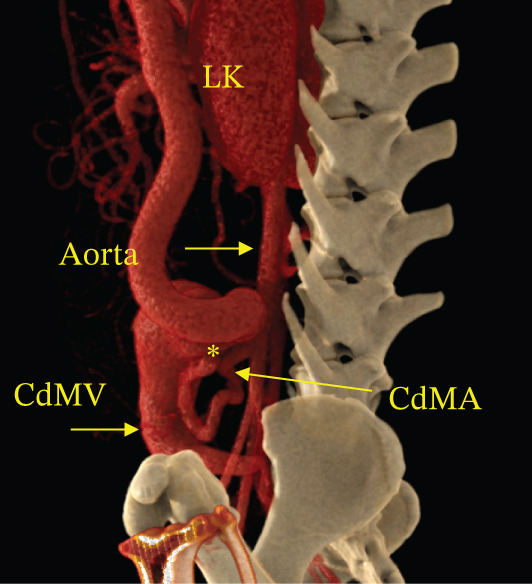
(b)

Based on these findings, the dog was diagnosed with portal hypertension secondary to a congenital caudal mesenteric arteriovenous fistula, with associated ascites and secondary acquired portosystemic shunts.

Surgical ligation of the arteriovenous fistula was performed under general anesthesia on Day 16. A ventral midline celiotomy was made from the xiphoid process to the cranial rim of the pubis. Exploration of the caudal abdomen revealed an abnormal vascular structure located dorsal to the colon, consistent with the suspected caudal mesenteric arteriovenous fistula (Figure 3a). Intraoperative portal venous pressure was measured via a mesenteric vein using a 24‐gauge over‐the‐needle catheter connected to an extension tube and pressure transducer. Portal venous pressure decreased from 23/20 (21) mmHg prior to occlusion to 19/16 (17) mmHg after occlusion. A temporary occlusion test was performed for approximately 5 min, during which no gross evidence of hemorrhage or intestinal congestion was observed. The caudal mesenteric artery at the fistulous communication was isolated and ligated proximal and distal to the communication using two circumferential ligatures of 0 braided nylon (polyamide) suture (Neobraid, Alfresa Pharma, Japan), each secured with square knots, and an additional titanium ligating clip was applied adjacent to the ligatures (Figure 3b). Intraoperatively, the liver appeared mildly ischemic, and a liver biopsy was obtained (Figure 3c). The spleen also had gross nodular changes suggestive of Gamna–Gandy nodules, which are commonly described in association with portal hypertension (Figure 3d).

Figure 3The caudal mesenteric artery was ligated proximally and distally to the fistulous communication using two circumferential ligatures (0 braided nylon [polyamide] suture) secured with square knots, with an additional titanium ligating clip applied adjacent to the ligatures (Day 16). (a) Gross appearance of a markedly dilated mesenteric vein in the caudal abdomen, consistent with venous congestion secondary to portal hypertension (arrow). (b) The shunting vessel at the fistulous site after occlusion at a single location using a 0 braided nylon ligature in combination with a titanium ligating clip. The occlusion site is indicated by the asterisk. (c) Gross appearance of the liver, which appeared mildly ischemic intraoperatively; a liver biopsy was obtained. (d) Gross appearance of the spleen showing multifocal dark, punctate capsular nodules consistent with Gamna–Gandy nodules, a finding often associated with portal hypertension.

**Figure:**
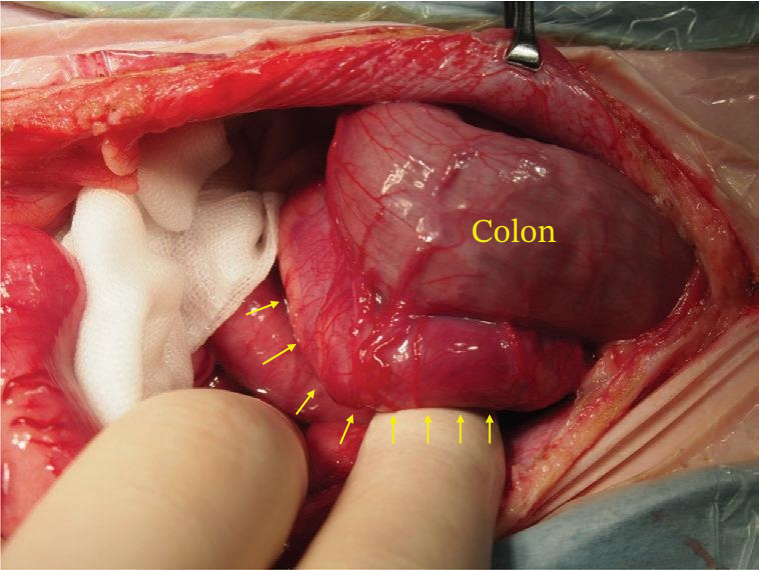
(a)

**Figure:**
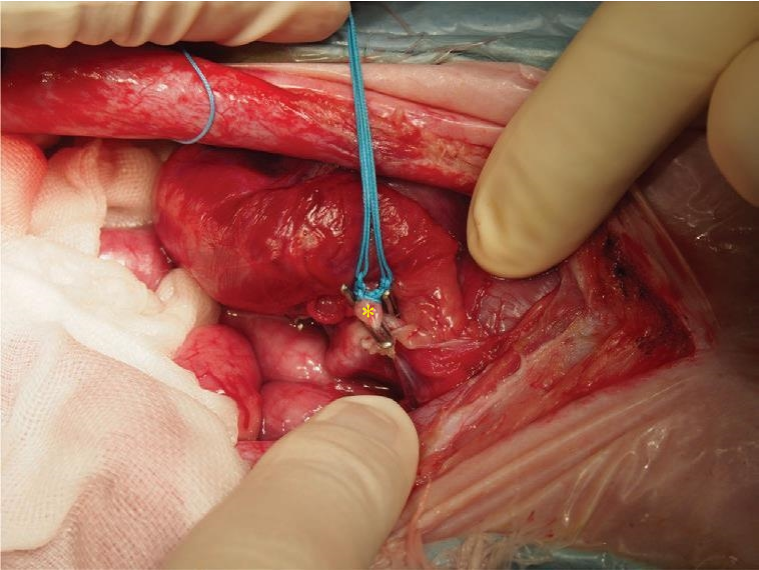
(b)

**Figure:**
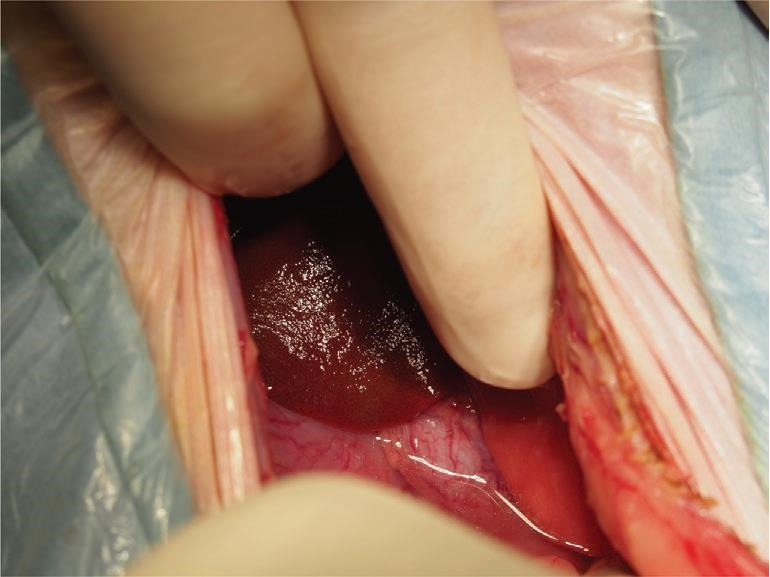
(c)

**Figure:**
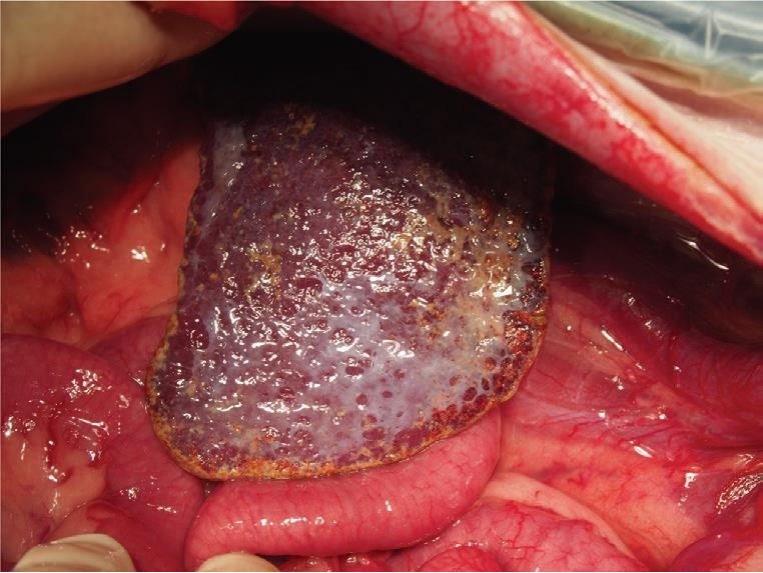
(d)

**Figure 4:**
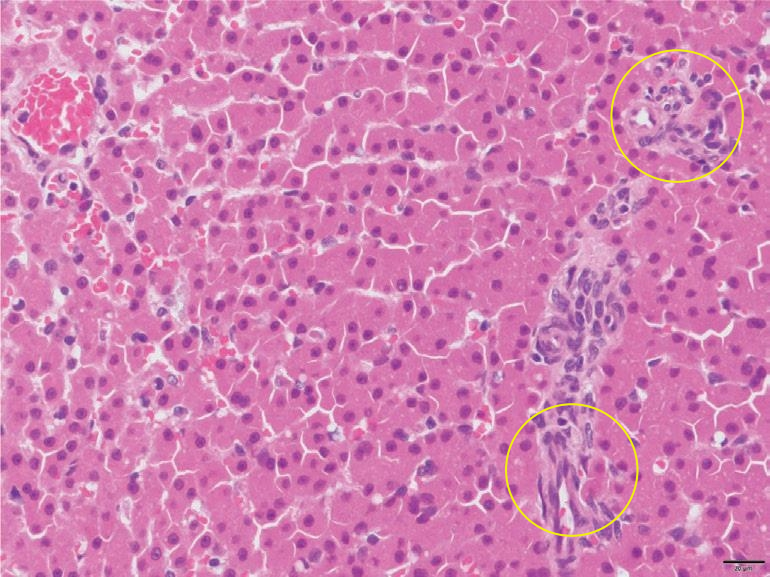
Photomicrograph of a liver biopsy specimen stained with hematoxylin and eosin (H&E), original magnification ×400. Portal vein hypoplasia is present, characterized by poorly developed/inconspicuous portal vein profiles within portal areas (yellow circles).

Histopathological examination of the liver biopsy specimen revealed hypoplastic and/or inconspicuous portal veins in several portal areas, consistent with portal vein hypoplasia and reduced portal triads (Figure 4).

**Figure 5:**
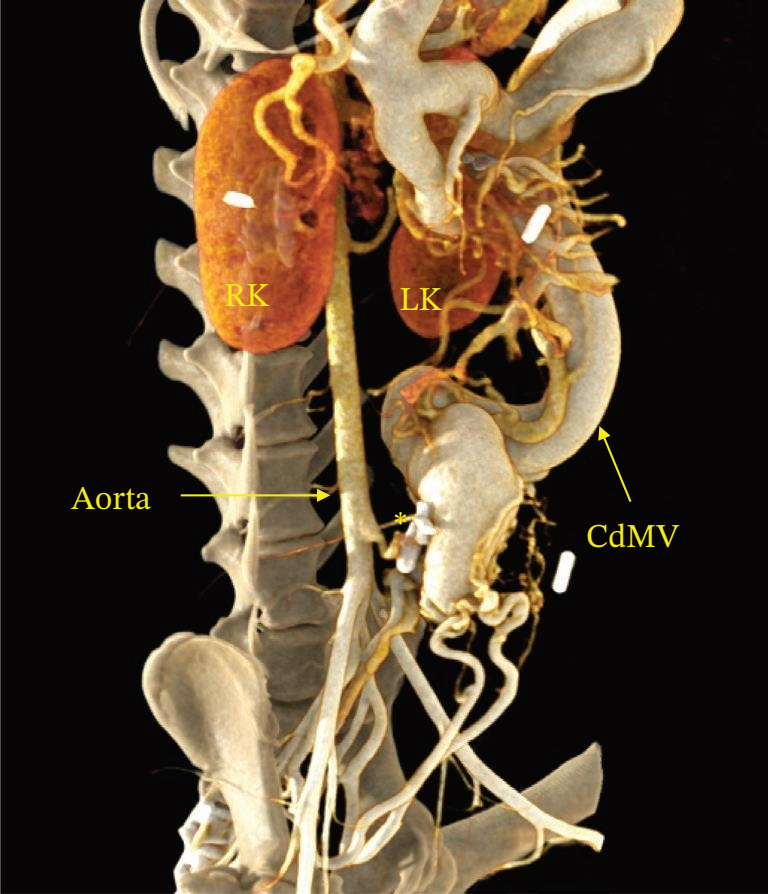
Follow‐up computed tomographic angiography obtained on Day 1191. A three‐dimensional volume‐rendered computed tomographic angiography image demonstrates persistent dilation of the caudal mesenteric vein; however, no evidence of residual arteriovenous shunting is identified at the previously diagnosed fistulous site, consistent with sustained occlusion. The asterisk indicates the site of occlusion. Abbreviations: CdMV = caudal mesenteric vein, RK = right kidney, LK = left kidney.

Postoperatively, the dog’s clinical condition improved, and ascites resolved by Postoperative Day 3. The dog was discharged on Day 22 (Postoperative Day 6). The dog remained clinically stable thereafter; however, ascites recurred on Day 868. On Day 1191, a follow‐up CT was performed (Figure 5). Dilation of the caudal mesenteric vein persisted; however, interruption of blood flow at the fistulous communication was confirmed, indicating successful occlusion. The dog was maintained on lactulose (0.3 mL/kg, PO, BID) and furosemide (1 mg/kg, PO, BID). Although intermittent abdominocentesis was still required approximately once monthly, hyperammonemia was not marked (NH_3_, 132 *μ*g/dL), and the dog’s general condition remained good.

## 3. Discussion

This report describes a dog with portal hypertension and ascites secondary to a congenital caudal mesenteric arteriovenous fistula. Surgical ligation of the abnormal arteriovenous communication resulted in an immediate decrease in portal venous pressure and rapid resolution of ascites. These findings support surgical interruption of mesenteric arteriovenous shunting as a potential treatment option in dogs [1–3].

Mesenteric arteriovenous fistulas can cause clinically important hemodynamic disturbances by directing arterial blood into the portal venous system, thereby increasing portal venous inflow and resulting in portal hypertension. Reported consequences include ascites, multiple acquired portosystemic shunts, and metabolic abnormalities such as hyperammonemia [3, 4, 6]. In the present case, intraoperative splenic changes consistent with Gamna–Gandy nodules supported chronic portal hypertension [8].

Treatment options for hepatic and mesenteric arteriovenous fistulas include surgical complete closure and, in selected cases, minimally invasive approaches such as endovascular embolization [1, 2, 9]. Regardless of the approach, assessment of portal hemodynamics is clinically relevant because abrupt alterations in portal blood flow may precipitate intestinal congestion or other complications. Portal venous pressure was monitored before and after occlusion of the shunting vessel, and ascites resolved by Postoperative Day 3. The dog was discharged on Postoperative Day 6 (Day 22), suggesting short‐term efficacy and perioperative safety of surgical ligation in this case.

Despite confirmation of sustained occlusion of the fistulous communication on follow‐up CT angiography, ascites recurred later in the follow‐up period (Day 868). Several factors may have contributed to recurrence. First, chronic portal hypertension and long‐standing remodeling of the splanchnic and portal venous vasculature may persist even after elimination of the primary shunt, as suggested by residual dilation of the caudal mesenteric vein. Second, histopathological examination of the liver biopsy revealed portal vein hypoplasia with reduced portal triads (Figure 4), suggesting concurrent hepatic vascular or microvascular abnormalities that could limit portal perfusion and predispose to ongoing portal hypertension [10]. Third, acquired portosystemic shunts may continue to influence portal flow distribution and hepatic function even after the initiating cause is corrected. In the present case, intermittent abdominocentesis was still required approximately once monthly through Day 1191; however, hyperammonemia was not marked at the time of follow‐up (NH_3_, 132 *μ*g/dL), and the dog’s general condition remained good under adjunct medical management with lactulose and furosemide. Collectively, these findings suggest that surgical ligation can provide meaningful clinical improvement and quality‐of‐life benefits, although long‐term monitoring and supportive management may still be required in some dogs.

Mesenteric arteriovenous fistulas are extremely rare in dogs, and previously reported cases have involved the cranial mesenteric vasculature. Notably, among the previously reported canine cases, two of three were Pembroke Welsh Corgis [1, 2]. Because the present dog was also a Pembroke Welsh Corgi, a breed predisposition or genetic contribution could be considered; however, no genetic investigations were performed in this case, and the available evidence remains insufficient to draw firm conclusions.

This report is limited by the description of a single case; therefore, conclusions regarding optimal treatment selection and long‐term prognosis should be drawn cautiously. In addition, portal venous pressure was assessed only intraoperatively, and serial quantitative assessment of portal hemodynamics during follow‐up was not performed. Further studies involving additional cases are warranted to better define the spectrum of clinical presentations, refine diagnostic and therapeutic strategies, and clarify long‐term outcomes after surgical or interventional treatment of mesenteric arteriovenous fistulas in dogs.

In conclusion, surgical ligation of a congenital caudal mesenteric arteriovenous fistula resulted in an immediate reduction in portal venous pressure and rapid resolution of ascites in this dog. Although ascites recurred later, sustained occlusion of the fistulous site was confirmed on follow‐up CT angiography, and long‐term general condition remained good with adjunct medical management. These findings support surgical intervention as a feasible treatment option for dogs with portal hypertension and ascites associated with congenital caudal mesenteric arteriovenous fistulas.

## Funding

No funding was received for this manuscript.

## Consent

No written consent has been obtained from the patients, as there is no patient‐identifiable data included in this case report.

## Conflicts of Interest

The authors declare no conflicts of interest.

## Supporting information


**Supporting Information** Additional supporting information can be found online in the Supporting Information section. Video S1 Axial images from computed tomographic angiography demonstrating marked dilation of the caudal mesenteric vein.

## Data Availability

The data that support the findings of this study are available from the corresponding author upon reasonable request.
